# Barriers and enablers to the implementation of patient-reported outcome and experience measures (PROMs/PREMs): protocol for an umbrella review

**DOI:** 10.1186/s13643-024-02512-5

**Published:** 2024-03-26

**Authors:** Guillaume Fontaine, Marie-Eve Poitras, Maxime Sasseville, Marie-Pascale Pomey, Jérôme Ouellet, Lydia Ould Brahim, Sydney Wasserman, Frédéric Bergeron, Sylvie D. Lambert

**Affiliations:** 1 Ingram School of Nursing, Faculty of Medicine and Health Sciences, McGill University, 680 Rue Sherbrooke O #1800, Montréal, QC H3A 2M7 Canada; 2 Centre for Clinical Epidemiology, Lady Davis Institute for Medical Research, Sir Mortimer B. Davis Jewish General Hospital, CIUSSS West-Central Montreal, 3755 Chem. de la Côte-Sainte-Catherine, Montréal, QC H3T 1E2 Canada; 3 Department of Family Medicine and Emergency Medicine, Faculty of Medicine and Health Sciences, Université de Sherbrooke, 3001 12 Ave N Building X1, Sherbrooke, QC J1H 5N4 Canada; 4 Centre Intégré Universitaire de Santé Et de Services Sociaux (CIUSSS) du Saguenay-Lac-Saint-Jean du Québec, 930 Rue Jacques-Cartier E, Chicoutimi, QC G7H 7K9 Canada; 5 Faculty of Nursing, Université Laval, 1050 Av. de La Médecine, Québec, QC G1V 0A6 Canada; 6 Centre de Recherche en Santé Durable VITAM, CIUSSS de La Capitale-Nationale, 2480, Chemin de La Canardière, Quebec City, QC G1J 2G1 Canada; 7 Faculty of Medicine & School of Public Health, Université de Montréal, Pavillon Roger-Gaudry, 2900 Edouard Montpetit Blvd, Montreal, QC H3T 1J4 Canada; 8 Centre de Recherche du Centre Hospitalier de L, Université de Montréal (CR-CHUM), 900 Saint Denis St., Montreal, QC H2X 0A9 Canada; 9 Direction of Nursing, CIUSSS de L’Ouest de L’Île-de-Montréal, 3830, Avenue Lacombe, Montreal, QC H3T 1M5 Canada; 10 Université Laval Library, Pavillon Alexandre-Vachon 1045, Avenue de La Médecine, Québec, Québec) G1V 0A6 Canada; 11 St. Mary’s Research Centre, CIUSSS de L’Ouest de L’Île-de-Montréal, 3777 Jean Brillant St, Montreal, QC H3T 0A2 Canada

**Keywords:** Patient-reported outcome measures, Patient-reported experience measures, PROMs, PREMs, Implementation science, Umbrella review, Systematic review, Overview of reviews, Barriers, Facilitators

## Abstract

**Background:**

Patient-reported outcome and experience measures (PROMs and PREMs, respectively) are evidence-based, standardized questionnaires that can be used to capture patients’ perspectives of their health and health care. While substantial investments have been made in the implementation of PROMs and PREMs, their use remains fragmented and limited in many settings. Analysis of multi-level barriers and enablers to the implementation of PROMs and PREMs has been hampered by the lack of use of state-of-the-art implementation science frameworks. This umbrella review aims to consolidate available evidence from existing quantitative, qualitative, and mixed-methods systematic and scoping reviews covering factors that influence the implementation of PROMs and PREMs in healthcare settings.

**Methods:**

An umbrella review of systematic and scoping reviews will be conducted following the guidelines of the Joanna Briggs Institute (JBI). Qualitative, quantitative, and mixed methods reviews of studies focusing on the implementation of PROMs and/or PREMs in all healthcare settings will be considered for inclusion. Eight bibliographical databases will be searched. All review steps will be conducted by two reviewers independently. Included reviews will be appraised and data will be extracted in four steps: (1) assessing the methodological quality of reviews using the JBI Critical Appraisal Checklist; (2) extracting data from included reviews; (3) theory-based coding of barriers and enablers using the Consolidated Framework for Implementation Research (CFIR) 2.0; and (4) identifying the barriers and enablers best supported by reviews using the Grading of Recommendations Assessment, Development and Evaluation-Confidence in the Evidence from Reviews of Qualitative research (GRADE-CERQual) approach. Findings will be presented in diagrammatic and tabular forms in a manner that aligns with the objective and scope of this umbrella review, along with a narrative summary.

**Discussion:**

This umbrella review of quantitative, qualitative, and mixed-methods systematic and scoping reviews will inform policymakers, researchers, managers, and clinicians regarding which factors hamper or enable the adoption and sustained use of PROMs and PREMs in healthcare settings, and the level of confidence in the evidence supporting these factors. Findings will orient the selection and adaptation of implementation strategies tailored to the factors identified.

**Systematic review registration:**

PROSPERO CRD42023421845.

**Supplementary Information:**

The online version contains supplementary material available at 10.1186/s13643-024-02512-5.

## Background

Capturing patients’ perspectives of their health and healthcare needs using standardized patient-reported outcome and experience measures (referred to herein as PROMs and PREMs, respectively) has been the focus of over 40 years of research [1, 2]. PROMs/PREMs are standardized, validated questionnaires (generic or disease-specific); PROMs are completed by patients about their health, functioning, and quality of life, whereas PREMs are focused on patients’ experiences whilst receiving care [1]. PROMs/PREMs are associated with a robust evidence-base across multiple illnesses; they can increase charting of patients’ needs [3], and improve patient-clinician communication [3–5], which in turn can lead to improved symptom management [4–6], thereby improving patients’ quality of life, reducing health care utilization [5], and increasing survival rates [7].

Multipurpose applications of PROMs/PREMs have led to substantial investments in their implementation. In the USA, PROMs are part of payer mandates; in the United Kingdom, they are used for benchmarking and included in a national registry; and Denmark has embedded them across healthcare sectors [8–11]. In Canada, the Canadian Institute for Health Information (CIHI) has advocated for a standardized core set of PROMs [12], and the Canadian Partnership Against Cancer (CPAC) recently spearheaded PROM implementation in oncology in 10 provinces/territories. In 2017, the Organisation for Economic Co-operation and Development (OECD) launched the Patient-Reported Indicators Surveys (PaRIS) to build international capacity for PROMs/PREMs in primary care [13]. Yet, in many countries across the globe, their use remains fragmented, characterized by broad swaths of pre-implementation, pilots, and full implementation in narrow domains [12, 14, 15]. PROM/PREM implementation remains driven by silos of local healthcare networks [16].

Barriers and enablers to the implementation of PROMs/PREMs exist at the patient level (e.g., low health literacy), [17] clinician level (e.g., obtaining PROM/PREM results from external digital platforms) [17–19], service level (e.g., lack of integration in clinics’ workflow) [17, 20] and organizational/system-level (e.g., organizational policies conflicting with PROM implementation goals) [21]. Foster and colleagues [22] conducted an umbrella review on the barriers and facilitators to implementing PROMs in healthcare settings. The umbrella review identified a number of bidirectional factors arising at different stages that can impact the implementation of PROMs; these factors were related to the implementation process, the organization, and healthcare providers [22]. However, the umbrella review focused solely on PROMs, excluding PREMs, and the theory-based analysis of implementation factors was limited. Another ongoing umbrella review is restricted to investigating barriers and enablers at the healthcare provider level, omitting the multilevel changes required for successful PROM/PREM implementation [23].

State-of-the-art approaches from implementation science can support the identification of multilevel factors influencing the implementation of PROMs and PREMs in different healthcare settings [24–26]. The second version of the Consolidated Framework for Implementation Research (CFIR 2.0) can guide the exploration of determinants influencing the implementation of PROMs and PREMs [27]. The CFIR is a meta-theoretical framework providing a repository of standardized implementation-related constructs at the individual, organizational, and external levels that can be applied across the spectrum of implementation research [27]. CFIR 2.0 includes five domains pertaining to the characteristics of the innovation targeted for implementation, the implementation process, the individuals involved in the implementation, the inner setting, and the outer setting [27]. Using an implementation framework to identify the multilevel factors influencing the implementation of PROMs/PREMs is critical to select and tailor implementation strategies to address barriers [28–31]. Implementation strategies are the “how”, the specific means or methods for promoting the adoption of evidence-based innovations (e.g., role revisions, audit, provide feedback) [32]. Selecting and adapting implementation strategies to facilitate the implementation of PROMs/PREMs can be time-consuming, as there are more than 73 implementation strategies to choose from [33]. Thus, a detailed understanding of the barriers to PROM/PREM implementation can inform and streamline the selection and adaptation of implementation strategies, saving financial, human, and material resources [24–26, 32, 34].

### Review objective and questions

In this umbrella review, we aim to consolidate available evidence from existing quantitative, qualitative, and mixed-methods systematic and scoping reviews covering factors that influence the implementation of PROMs and PREMs in healthcare settings.

We will address the following questions:

1. What are the factors that hinder or enable the implementation of PROMs and PREMs in healthcare settings, and what is the level of confidence in the evidence supporting these factors?
2. What are the similarities and differences in barriers and enablers across settings and geographical regions?
3. What are the similarities and differences in the perceptions of barriers and enablers between patients, clinicians, managers, and decision-makers?
4. What are the implementation theories, models, and frameworks that have been used to guide research in this field?

## Methods

### Review design and registration

An umbrella review of systematic and scoping reviews will be conducted following the guidelines of the Joanna Briggs Institute (JBI) [35, 36]. The umbrella review is a form of evidence synthesis that aims to address the challenge of collating, assessing, and synthesizing evidence from multiple reviews on a specific topic [35]. This protocol was registered on PROSPERO (CRD42023421845) and is presented according to the Preferred Reporting Items for Systematic Review and Meta-Analysis Protocols (PRISMA-P) guidelines (see Supplementary material 1) [37]. We will use the Preferred Reporting Items for Overviews of Reviews (PRIOR) guidelines [38] and the PRISMA guidelines [39] to report results (e.g., flowchart, search process).

### Eligibility criteria

The eligibility criteria were developed following discussions among the project team including researchers with experience in the implementation of PROMs and PREMs in different fields (e.g., cancer care, primary care) and implementation science. These criteria were refined after being piloted on a set of studies. The final eligibility criteria for the review are detailed in Table 1. We will consider for inclusion all qualitative, quantitative, and mixed methods reviews of studies focusing on the implementation of PROMs or PREMs in any healthcare setting.

**Table 1:** Eligibility criteria

Criterion	Inclusion	Exclusion
*Population (P)*	• Patients • Healthcare providers • Middle-level managers • Decision-makers • Policymakers	• Children and adolescents (< 18 years old) or their parents
*Phenomena of interest (I)*	• Factors (barriers, enablers) influencing the implementation of PROMs/PREMs • Experiences of implementing PROMs/PREMs • Views or attitudes towards PROMs/PREMs	• Impact or effectiveness of PREMs/PROMs • Measurement development, testing, and selection • Mechanisms by which PROMs/PREMs work
*Context (C)*	• Acute care settings (e.g., hospitals, emergency departments, oncology care centers, mental health facilities) • Outpatient care settings • Long-term care settings (e.g., nursing homes, rehabilitation centers, palliative care settings, hospice care facilities) • Home-based care settings • Primary care settings (e.g., clinics, community health centers)	• Pediatric and adolescent care settings
*Study design*	• Qualitative, quantitative, and mixed-methods systematic and scoping reviews	• Efficacy/effectiveness reviews • Psychometric reviews
*Language*	• Published in English or French	• Any other language
*Geographic*	• Any country	• None
*Time period*	• Published from the onset of each database to now	• None

### Information sources

Searches will be conducted in eight databases: CINAHL, via EBSCOhost (1980 to present); Cochrane Database of Systematic Reviews; Evidence-Based Medicine Reviews; EMBASE, via Ovid SP (1947 to present); ERIC, via Ovid SP (1966 to present); PsycINFO, via APA PsycNet (1967 to present); PubMed (including MEDLINE), via NCBI (1946 to present); Web of Science, via Clarivate Analytics (1900 to present). CINAHL is a leading database for nursing and allied health literature. The Cochrane Database of Systematic Reviews and Evidence-Based Medicine Reviews are essential for accessing high-quality systematic reviews and meta-analyses. EMBASE is a biomedical and pharmacological database offering extensive coverage of drug research, pharmacology, and medical devices, complementing PubMed. ERIC provides valuable insights from educational research that are relevant to our study given the intersection of healthcare and education in PROMs and PREMs. PsycINFO is crucial for accessing research on the psychological aspects of PROMs and PREMs. PubMed, encompassing MEDLINE, is a primary resource for biomedical literature. Web of Science offers a broad and diverse range of scientific literature providing interdisciplinary coverage. We will use additional strategies to complement our exploration including examining references cited in eligible articles, searching for authors who have published extensively in the field, and conducting backward/forward citation searches of related systematic reviews and influential articles.

### Search strategy

A comprehensive search strategy was developed iteratively by the review team in collaboration with an experienced librarian with a Master’s of Science in Information (FB). First, an initial limited search of MEDLINE and CINAHL will be undertaken to identify reviews on PROM/PREM implementation. The text words contained in the titles and abstracts, and the index terms used to describe these reviews will be analyzed and applied to a modified search strategy (as needed). We adapted elements from the search strategies of two recent reviews in the field of PROM/PREM implementation [22, 23] to fit our objectives. The search strategy for PubMed is presented in Supplementary material 2. The search strategy will be tailored for each information source. The complete search strategy for each database will be made available for transparency and reproducibility in the final manuscript.

### Selection process

All identified citations will be collated and uploaded into the Covidence systematic review software (Veritas Health Innovation, Melbourne, Australia), and duplicates removed. Following training on 50 titles, titles will be screened by two independent reviewers for assessment against the inclusion criteria for the review. Multiple rounds of calibration might be needed. Once titles have been screened, retained abstracts will be reviewed, preferably by the same two reviewers. However, inter-rater reliability will be re-established on 50 abstracts to re-calibrate (as needed). Lastly, the full texts of retained abstracts will be located and assessed in detail against the inclusion criteria by two independent reviewers. Reasons for excluding articles from full-text review onwards will be recorded in the PRIOR flow diagram (PRISMA-like flowchart) [38]. Any disagreements that arise between the reviewers at each stage of the selection process will be resolved through discussion, or with an additional reviewer. More specifically, throughout the project, weekly team meetings will be held and will provide the opportunity for the team to discuss and resolve any disagreement that arises during the different stages, from study selection to data extraction.

### Quality appraisal and data extraction

**Fig. 1:**
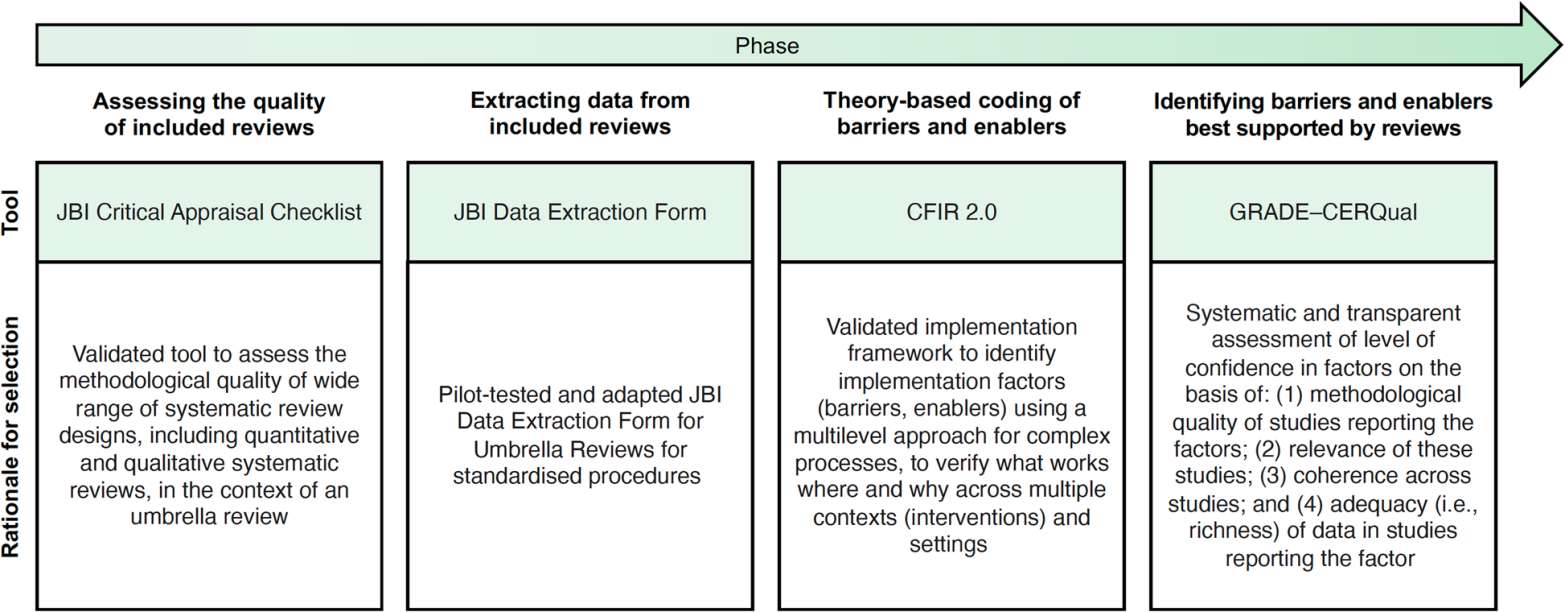
Tools/methodology applied in each phase of the umbrella review. Figure adapted from Boudewijns and colleagues [41] with permission. CFIR 2.0 = Consolidated Framework for Implementation Research, version 2 [27]. GRADE–CERQual = Grading of Recommendations Assessment Development and Evaluation–Confidence in the Evidence from Reviews of Qualitative Research [42]. JBI = Joanna Briggs Institute [36]

As presented in Fig. 1, included reviews will be appraised and data will be extracted and analyzed in four steps using validated tools and methodologies [27, 36, 40]. All four steps will be conducted by two reviewers independently, and a third will be involved in case of disagreement. More reviewers may be needed depending on the number of reviews included.

#### Step 1—assessing the quality of included reviews

In the first step, two reviewers will independently assess the methodological quality of the reviews using the JBI Critical Appraisal Checklist for Systematic Reviews and Research Syntheses, presented in Supplementary material 3. We have selected this checklist for its comprehensiveness, applicability to different types of knowledge syntheses, and ease of use, requiring minimal training for reviewers to apply it. The checklist consists of 11 questions. It evaluates whether the review question is clearly and explicitly stated, the inclusion criteria were appropriate for that question, and the search strategy and sources used to determine if they were suitable and adequate for capturing relevant studies. It also assesses the appropriateness of the criteria used for appraising studies, as well as whether the critical appraisal was conducted independently by two or more reviewers. The checklist further examines if there were methods in place to minimize errors during data extraction, if the methods used to combine studies were appropriate, and whether the likelihood of publication bias was assessed. Additionally, it verifies if the recommendations for policy and/or practice are supported by the reported data and if the directives for new research are appropriate. Each question should be answered as “yes”, “no”, or “unclear”. Not applicable “NA” is also provided as an option and may be appropriate in rare instances. The results of the quality appraisal will provide the basis for assessing confidence in the evidence in step four. Any disagreements that arise between the reviewers will be resolved through discussion, or with a third reviewer, or at team meetings.

#### Step 2—extracting data from included reviews

For the second step, we have developed a modified version of the JBI Data Extraction Form for Umbrella Reviews, presented in Supplementary material 3. We will pilot our data extraction form on two of the included reviews, and it will be revised for clarity, as needed. Subsequently, two independent reviewers will conduct all extraction for each review independently. We will collect the following data: (a) authors and date; (b) country; (c) review aims, objectives; (d) focus of the review; (e) context; (f) population; (g) eligibility criteria; (f) review type and methodology; (g) data sources; (h) dates of search; (i) number of included studies; (j) characteristics of included studies (including study type, critical appraisal score); (k) implementation framework guiding analysis; (l) implementation strategies discussed; (m) results and significance; and (n) conclusions. Barriers and enablers will be extracted separately in step 3. Any disagreements that arise between the reviewers will be resolved through discussion, or with a third reviewer, or at team meetings.

#### Step 3—theory-based coding of barriers and enablers

**Fig. 2:**
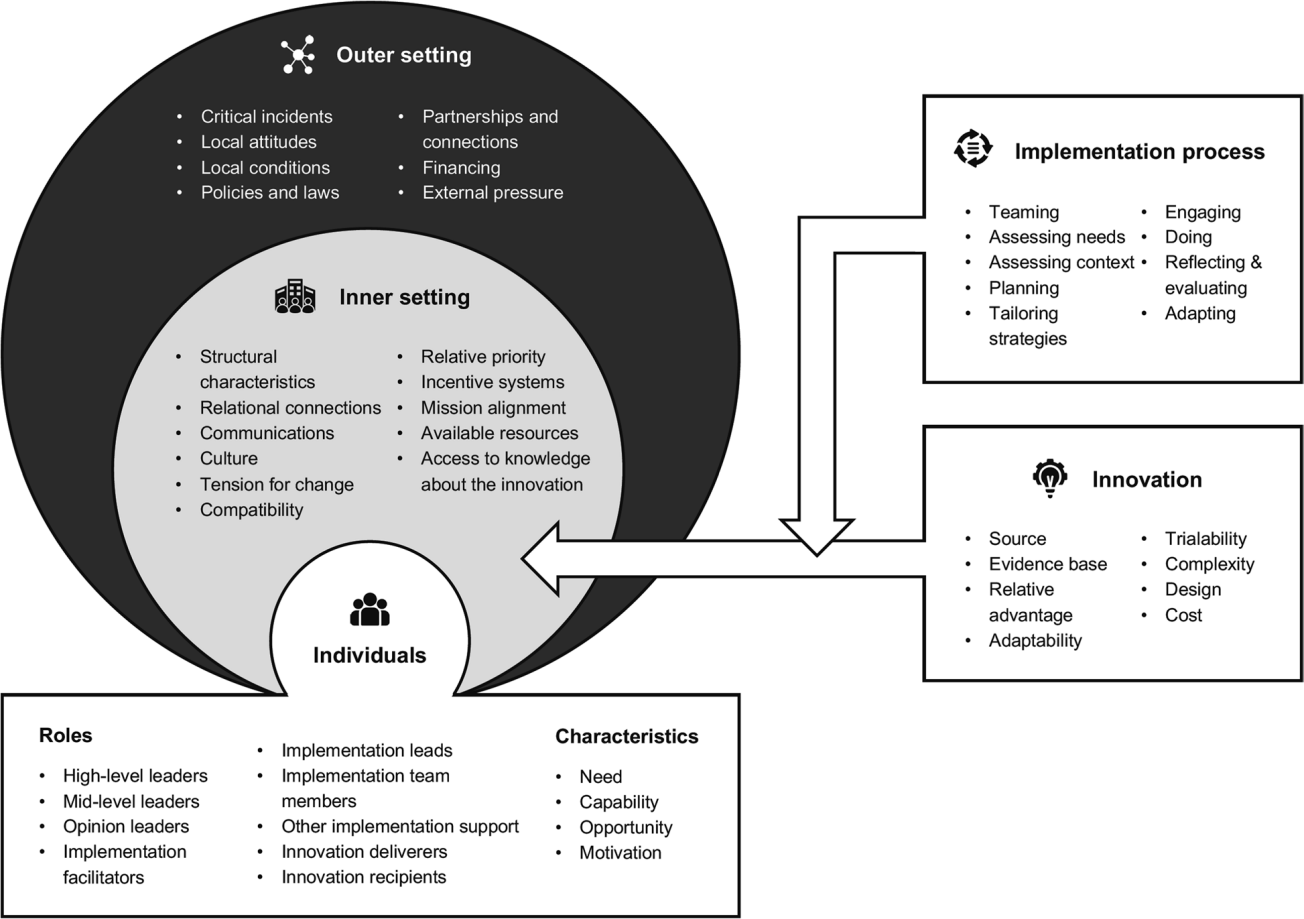
The second version of the Consolidated Framework for Implementation Research and its five domains: innovation, outer setting, inner setting, individuals, and implementation process [27, 43]

In the third step, we will use the second version of the Consolidated Framework for Implementation Research (CFIR) [27] to guide our proposed exploration of determinants influencing the implementation of PROMs and PREMs (see Fig. 2). The CFIR is a meta-theoretical framework providing a repository of standardized implementation-related constructs at the individual, organizational, and external levels that can be applied across the spectrum of implementation research. CFIR contains 48 constructs and 19 subconstructs representing determinants of implementation across five domains: *Innovation* (i.e., PROMs and PREMs), *Outer Setting* (e.g., national policy context), *Inner Setting* (e.g., work infrastructure), *Individuals* (e.g., healthcare professional motivation) and *Implementation Process* (e.g., assessing context) [27]. To ensure that coding remains grounded in the chosen theoretical framework, we have developed a codebook based on the second version of the CFIR, presented in Supplementary material 3. Furthermore, an initial training session and regular touchpoints will be held to discuss coding procedures among the team members involved.

To code factors influencing the implementation of PROMs and PREMs using the CFIR, we will upload all PDFs of the included reviews and their appendices in the NVivo qualitative data analysis software (QSR International, Burlington, USA). All reviews will be independently coded by two reviewers. Any disagreements that arise between the reviewers will be resolved through discussion, or with a third reviewer.

#### Step 4—identifying the barriers and enablers best supported by the reviews

In the fourth and final step, we will use the Grading of Recommendations Assessment, Development, and Evaluation-Confidence in the Evidence from Reviews of Qualitative research (GRADE-CERQual) approach to assess the level of confidence in the barriers and enablers to PROM/PREM implementation identified in step 3 (see Supplementary material 3). This process will identify which barriers and enablers are best supported by the evidence in the included reviews. GRADE-CERQual includes four domains: (a) methodological limitations, (b) coherence and (c) adequacy of data, and (d) relevance (see Table 2). For each review finding, we will assign a score per domain from one point (substantial concerns) to four points (no concerns to very minor concerns). The score for the methodological limitations of the review will be assigned based on the JBI Critical Appraisal (step 1). The score for coherence will be assigned based on the presence of contradictory findings as well as ambiguous/incomplete data for that finding in the umbrella review. The score for adequacy of data will be assigned based on the richness of the data supporting the umbrella review finding. Finally, the score for relevance will be assigned based on how well the included reviews supporting a specific barrier or enabler to the implementation of PROMs/PREMs are applicable to the umbrella review context. This will allow us to identify which factors are supported by evidence with the highest level of confidence, and their corresponding level of evidence. A calibration exercise will be conducted on three systematic reviews with team members involved in this stage of the umbrella review, and adjustments to procedures will be discussed in team meetings.

**Table 2:** Definitions of the components of the GRADE-CERQual for the context of this umbrella review (adapted from [44])

Component	Definition	Guiding questions
Methodological limitations	The extent to which there are concerns about the design or conduct of the reviews that contributed evidence to an individual umbrella review finding	What is the methodological quality of reviews supporting a specific barrier or enabler to the implementation of PROMs/PREMs?
Coherence	An assessment of how clear and cogent the fit is between the data from the reviews and an umbrella review finding that synthesizes that data	How clear and logical is the fit between a specific barrier or enabler to the implementation of PROMs/PREMs and the reviews supporting this factor?
Adequacy of data	An overall determination of the degree of richness and quantity of data supporting an umbrella review finding	What is the richness and quantity of the data supporting a specific barrier or enabler to the implementation of PROMs/PREMs?
Relevancy	The extent to which the body of evidence from the reviews supporting an umbrella review finding is applicable to the context (perspective or population, phenomenon of interest, setting) specified in the umbrella review question	How well do the reviews support a specific barrier or enabler to the implementation of PROMs/PREMs that apply to the umbrella review context?

The data synthesis plan for the umbrella review has been meticulously designed to present extracted data in a format that is both informative and accessible, aiding in decision-making and providing a clear overview of the synthesized evidence.

Data extracted from the included systematic reviews will be organized into diagrams and tables, ensuring the presentation is closely aligned with our objectives and scope. These will categorize the distribution of reviews in several ways: by the year or period of publication, country of origin, target population, context, type of review, and various implementation factors. This stratification will allow for an at-a-glance understanding of the breadth and focus of the existing literature. To further assist in the application of the findings, a Summary of Qualitative Findings (SoQF) table will be constructed. This table will list each barrier and enabler identified within the systematic reviews and provide an overall confidence assessment for each finding. The confidence assessment will be based on the methodological soundness and relevance of the evidence supporting each identified barrier or enabler. Importantly, the SoQF table will include explanations for these assessments, making the basis for each judgement transparent [42]. Additionally, a CERQual Evidence Profile will be prepared, offering a detailed look at the reviewers’ judgements concerning each component of the CERQual approach. These components contribute to the overall confidence in the evidence for each identified barrier or enabler. The CERQual Evidence Profile will serve as a comprehensive record of the quality and applicability of the evidence [42].

Finally, we will conduct a narrative synthesis accompanying the tabular and diagrammatic presentations, summarizing the findings and discussing their implications concerning the review’s objectives and questions. This narrative will interpret the significance of the barriers and enablers identified, explaining how the synthesized evidence fits into the existing knowledge base and pointing out potential directions for future research or policy formulation.

## Discussion

This protocol outlines an umbrella review aiming to consolidate available evidence on the implementation of PROMs and PREMs in healthcare settings. Through our synthesis of quantitative, qualitative, and mixed-methods systematic and scoping reviews, we will answer two key questions: which factors hinder or enable the adoption and sustained use of PROMs and PREMs in healthcare settings, and what is the level of confidence in the evidence supporting these factors? Our findings will indicate which factors can influence the adoption of PROMs and PREMs, including clinician buy-in, patient engagement, and organizational support. Furthermore, our review will provide key insights regarding how barriers and enablers to PROM/PREM implementation differ across settings and how perceptions around their implementation differ between patients, clinicians, managers, and decision-makers. The consideration of different healthcare settings and the inclusion of studies from different geographical regions and healthcare systems will provide a global perspective, essential for understanding how context-specific factors might influence the generalizability of findings.

Strengths of this umbrella review include the use of a state-of-the-art implementation framework (CFIR 2.0) to identify, categorize, and synthesize multilevel factors influencing the implementation of PROMs/PREMS, and the use of the GRADE-CERQual approach to identify the level of confidence in the evidence supporting these factors. Using CFIR 2.0 will address a key limitation of current research in the field, since reviews and primary research are often focused on provider- and patient-level barriers and enablers, omitting organizational- and system-level factors affecting PROM/PREM implementation. This umbrella review will expose knowledge gaps to orient further research to improve our understanding of the complex factors at play in the adoption and sustained use of PROMs and PREMs in healthcare settings. Importantly, using CFIR 2.0 will allow the mapping of barriers and enablers identified to relevant implementation strategy taxonomies, such as the Expert Recommendations for Implementing Change (ERIC) Taxonomy [34]. This is crucial for designing tailored implementation strategies, as it can ensure that the chosen approaches to support implementation are directly aligned with the specific barriers and enablers to the uptake of PROMs and PREMs.

Umbrella reviews are also associated with some limitations, including being limited to the inclusion of systematic reviews and other knowledge syntheses, while additional primary studies are likely to have since been published. These additional empirical studies will not be captured, but we will minimize this risk by updating the search strategy at least once before the completion of the umbrella review. A second key challenge in umbrella reviews is the overlap between the primary studies, as many studies will have been included in different systematic reviews on the same topic. To address this issue, we will prepare a matrix of primary studies included in systematic reviews to gain insight into double counting of primary studies.

We will maintain an audit trail document amendments to this umbrella review protocol and report these in both the PROSPERO register and subsequent publications. Findings will be disseminated through publications in peer-reviewed journals in the fields of implementation, medicine, as well as health services, and policy research. We will also disseminate results through relevant conferences and social media using different strategies (e.g., graphical abstract). Furthermore, we will leverage existing connections between SDL and decision-makers at a provincial and national level in Canada to disseminate the findings of the review to a wider audience (e.g., the Director of Quebec Cancerology Program, Canadian Association of Psychosocial Oncology).

### Supplementary Information

**Supplementary Material 1.**

**Supplementary Material 2.**

**Supplementary Material 3.**

## Data Availability

Data sharing is not applicable to this article as no datasets were generated or analyzed for the purposes of this publication.

## Abbreviations

CERQual: Confidence in the evidence from reviews of qualitative research
CFIR: Consolidated framework for implementation research
CIHI: Canadian Institute for Health Information
CPAC: Canadian Partnership Against Cancer
ERIC: Expert recommendations for implementing change
GRADE: Grading of recommendations assessment, development and evaluation
JBI: Joanna Briggs Institute
OECD: Organisation for economic co-operation and development
PaRIS: Patient-reported indicators surveys
PREM: Patient-reported experience measure
PRIOR: Preferred reporting items for overviews of reviews
PRISMA: Preferred reporting items for systematic review and meta-analysis
PRISMA-P: Preferred reporting items for systematic review and meta-analysis protocols
PROM: Patient-reported outcome measure

## References

[1] Kingsley C, Patel S (2017). Patient-reported outcome measures and patient-reported experience measures. BJA Education.

[2] Jamieson Gilmore K CI, Coletta L, Allin S. The uses of patient reported experience measures in health systems: a systematic narrative review. Health Policy. 2022. 10.1016/j.healthpol.2022.07.008 35934546

[3] Gibbons CPI, Gonçalves-Bradley DC (2021). Routine provision of feedback from patientreported outcome measurements to healthcare providers and patients in clinical practice. Cochrane Database Syst Rev..

[4] Howell DMS, Wilkinson K (2015). Patient-reported outcomes in routine cancer clinical practice: a scoping review of use, impact on health outcomes, and implementation factors. Ann Oncol.

[5] Kotronoulas GKN, Maguire R (2014). What is the value of the routine use of patient-reported outcome measures toward improvement of patient outcomes, processes of care, and health service outcomes in cancer care? A systematic review of controlled trials. J Clin Oncol.

[6] Chen J OL, Hollis SJ. A systematic review of the impact of routine collection of patient reported outcome measures on patients, providers and health organisations in an oncologic setting. BMC Health Serv Res. 2013;13(211). 10.1186/1472-6963-13-211 PMC3700832 23758898

[7] Basch E (2016). Symptom monitoring With patient-reported outcomes during routine cancer treatment: A randomized controlled trial. J Clin Oncol.

[8] Forcino RCMM, Engel JA, O'Malley AJ, Elwyn G (2020). Routine patient-reported experience measurement of shared decision-making in the USA: a qualitative study of the current state according to frontrunners. BMJ Open.

[9] Timmins N (2008). NHS goes to the PROMS. BMJ.

[10] Mjåset C. Value-based health care in four different health care systems. NEJM Catalyst. 2020.

[11] Sekretariatet P. PRO – patient reported outcome. https://pro-danmark.dk/da/proenglish.

[12] Terner MLK, Chow C, Webster G (2021). Advancing PROMs for health system use in Canada and beyond. J Patient Rep Outcomes.

[13] Slawomirski L, van den Berg M, Karmakar-Hore S (2018). Patient-Reported indicator survey (Paris): aligning practice and policy for better health outcomes. World Med J.

[14] Ahmed SBL, Bartlett SJ (2020). A catalyst for transforming health systems and person-centred care: Canadian national position statement on patient-reported outcomes. Curr Oncol.

[15] Pross C, Geissler A, Busse R (2017). Measuring, reporting, and rewarding quality of care in 5 nations: 5 policy levers to enhance hospital quality accountability. Milbank Q.

[16] Ernst SCK, Steinbeck V, Busse R, Pross C (2022). Toward system-wide implementation of patient-reported outcome measures: a framework for countries, states, and regions. Value in Health.

[17] Nguyen HBP, Dhillon H, Sundaresan P (2021). A review of the barriers to using Patient-Reported Outcomes (PROs) and Patient-Reported Outcome Measures (PROMs) in routine cancer care. J Med Radiation Sci.

[18] Davis SAM, Smith M (2022). Paving the way for electronic patient-centered measurement in team-based primary care: integrated knowledge translation approach. JMIR Form Res.

[19] Bull CTH, Watson D, Callander EJ (2022). Selecting and implementing patient-reported outcome and experience measures to assess health system performance. JAMA Health Forum.

[20] Schepers SAHL, Zadeh S, Grootenhuis MA, Wiener L (2016). Healthcare professionals' preferences and perceived barriers for routine assessment of patient-reported outcomes in pediatric oncology practice: moving toward international processes of change. Pediatr Blood Cancer.

[21] Glenwright BG, Simmich J, Cottrell Mea. Facilitators and barriers to implementing electronic patient-reported outcome and experience measures in a health care setting: a systematic review. J Patient Rep Outcomes. 2023;7(13). 10.1186/s41687-023-00554-2 10.1186/s41687-023-00554-2 PMC9928985 36786914

[22] Foster A, Croot L, Brazier J, Harris J, O’Cathain A (2018). The facilitators and barriers to implementing patient reported outcome measures in organisations delivering health related services: a systematic review of reviews. J Patient Rep Outcomes.

[23] Wolff AC, Dresselhuis A, Hejazi Sea. Healthcare provider characteristics that influence the implementation of individual-level patient-centered outcome measure (PROM) and patient-reported experience measure (PREM) data across practice settings: a protocol for a mixed methods systematic review with a narrative synthesis. Syst Rev. 2021;10(169). 10.1186/s13643-021-01725-2 10.1186/s13643-021-01725-2 PMC8188663 34108024

[24] Grimshaw JM, Eccles MP, Lavis JN, Hill SJ, Squires JE (2012). Knowledge translation of research findings. Implement Sci.

[25] French SD, Green SE, O’Connor DA (2012). Developing theory-informed behaviour change interventions to implement evidence into practice: a systematic approach using the Theoretical Domains Framework. Implement Sci.

[26] Wolfenden L, Foy R, Presseau J, Grimshaw J M, Ivers N M, al. PBJe. Designing and undertaking randomised implementation trials: guide for researchers. BMJ. 2021;372. 10.1136/bmj.m3721 10.1136/bmj.m3721 PMC7812444 33461967

[27] Damschroder LJ, Reardon, C.M., Widerquist, M.A.O. et al. ,. The updated Consolidated Framework for Implementation Research based on user feedback. Implementation Science. 2022;17:75.10.1186/s13012-022-01245-0 10.1186/s13012-022-01245-0 PMC9617234 36309746

[28] Bradshaw ASM, Mulderrig M (2021). Implementing person-centred outcome measures in palliative care: An exploratory qualitative study using Normalisation Process Theory to understand processes and context. Palliat Med.

[29] Stover AMHL, van Oers HA, Greenhalgh J, Potter CM (2021). Using an implementation science approach to implement and evaluate patient-reported outcome measures (PROM) initiatives in routine care settings. Qual Life Res.

[30] Manalili KSM (2021). Using implementation science to inform the integration of electronic patient-reported experience measures (ePREMs) into healthcare quality improvement: description of a theory-based application in primary care. Qual Life Res.

[31] Patey AM, Fontaine, G., Francis, J. J., McCleary, N., Presseau, J., & Grimshaw, J. M. Healthcare professional behaviour: health impact, prevalence of evidence-based behaviours, correlates and interventions. Psychol Health. 2022:766–794.10.1080/08870446.2022.2100887 10.1080/08870446.2022.2100887 35839082

[32] Proctor EK, Powell BJ, McMillen JC (2013). Implementation strategies: recommendations for specifying and reporting. Implement Sci.

[33] Powell BJ, Waltz TJ, Chinman MJ (2017). A refined compilation of implementation strategies: results from the Expert Recommendations for Implementing Change (ERIC) project. Implementation Sci.

[34] Waltz TJ, Powell BJ, Matthieu MM, et al. Use of concept mapping to characterize relationships among implementation strategies and assess their feasibility and importance: results from the Expert Recommendations for Implementing Change (ERIC) study. Implement Sci. 2015;10:109. 10.1186/s13012-015-0295-0. 10.1186/s13012-015-0295-0 PMC4527340 26249843

[35] Aromataris E MZ. Chapter 11: Umbrella Reviews. In: Aromataris E, Munn Z, eds. Joanna Briggs Institute Reviewer's Manual. The Joanna Briggs Institute; 2020.

[36] Aromataris E, Fernandez R, Godfrey C, Holly C, Kahlil H, Tungpunkom P (2015). Summarizing systematic reviews: methodological development, conduct and reporting of an Umbrella review approach. Int J Evid Based Healthc.

[37] Moher D, Shamseer, L., Clarke, M. et al. Preferred reporting items for systematic review and meta-analysis protocols (PRISMA-P) 2015 statement. Syst Rev. 2015;4(1). 10.1186/2046-4053-4-1 10.1186/2046-4053-4-1 PMC4320440 25554246

[38] Gates MGA, Pieper D, Fernandes RM, Tricco AC, Moher D (2022). Reporting guideline for overviews of reviews of healthcare interventions: development of the PRIOR statement. BMJ.

[39] Page MJ, McKenzie JE, Bossuyt PM, Boutron I, Hoffmann TC, Mulrow CD, Moher D. The (2020). The PRISMA 2020 statement: an updated guideline for reporting systematic reviews. Int J Surg..

[40] Dixon-Woods M, Agarwal, S., Young, B., Jones, D., & Sutton, A. Integrative approaches to qualitative and quantitative evidence. Health Development Agency; 2004. 10.1177/135581960501000110 15667704

[41] Boudewijns EA, Trucchi, M., van der Kleij, R. M., Vermond, D., Hoffman, C. M., Chavannes, N. H., ... & Brakema, E. A. Facilitators and barriers to the implementation of improved solid fuel cookstoves and clean fuels in low-income and middle-income countries: an umbrella review. Lancet Planet Health. 2022. 10.1016/S2542-5196(22)00094-8 35716672

[42] Lewin SGC, Munthe-Kaas H (2015). Using qualitative evidence in decision making for health and social interventions: an approach to assess confidence in findings from qualitative evidence syntheses (GRADE-CERQual). PLoS Med.

[43] The Centre for Implementation. The Consolidated Framework for Implementation Research (CFIR) 2.0. Adapted from "The updated Consolidated Framework for Implementation Research based on user feedback," by Damschroder, L.J., Reardon, C.M., Widerquist, M.A.O. et al., 2022, Implementation Sci 17, 75. Image copyright 2022 by The Center for Implementation. https://thecenterforimplementation.com/toolbox/cfir 10.1186/s13012-022-01245-0 PMC9617234 36309746

[44] Lewin S, Booth A, Glenton C, et al. Applying GRADE-CERQual to qualitative evidence synthesis findings: introduction to the series. Implementation Sci 2018;13(Suppl 1):2. 10.1186/s13012-017-0688-3. 10.1186/s13012-017-0688-3 PMC5791040 29384079

