# Supplementary material for: Barriers and enablers to the implementation of patient-reported outcome and experience measures (PROMs/PREMs): protocol for an umbrella review
Source: Syst Rev. 2024 Mar 26;13:96. doi: 10.1186/s13643-024-02512-5 (PMC10964633; doi:10.1186/s13643-024-02512-5)
Supplement: Supplementary file 2 — Supplementary Material 2. [file 13643_2024_2512_MOESM2_ESM.docx]

**Supplementary Material 2. PubMed search strategy.**

May 24, 2023

| **#** | **Search terms** | **Results** |
| --- | --- | --- |
| #1 | (PROM[Title]) OR (PROMs[Title]) OR (Patient-reported outcome measure*[Title]) OR (PREM[Title]) OR (PREMs[Title]) OR (Patient-reported experience measure*[Title]) OR (PRO[Title]) OR (PROS[Title]) OR (EPRO[Title]) OR (EPROS[Title]) OR (patient reported outcom*[Title]) OR (distress screening[Title]) OR (screening for distress[Title] OR exp/ Patient Reported Outcome Measures) | 42,832 |
| #2 | (review[Title]) OR (evidence synthesis[Title]) OR (qualitative synthesis[Title/Abstract]) OR (knowledge synthesis[Title/Abstract]) OR (systematic review[Title/Abstract]) OR (scoping review[Title/Abstract]) OR (systematic literature review[Title/Abstract]) | 731,193 |
| #3 | #1 AND #2 | **1,521** |
