# Supplementary material for: Barriers and enablers to the implementation of patient-reported outcome and experience measures (PROMs/PREMs): protocol for an umbrella review
Source: Syst Rev. 2024 Mar 26;13:96. doi: 10.1186/s13643-024-02512-5 (PMC10964633; doi:10.1186/s13643-024-02512-5)
Supplement: Supplementary file 3 — Supplementary Material 3. [file 13643_2024_2512_MOESM3_ESM.docx]

**Supplementary material 3**

**Barriers and Enablers to the Implementation of Patient-Reported Outcome and Experience Measures (PROMs/PREMs): Protocol for an Umbrella Review**

**Content**

[1. JBI Critical Appraisal Checklist for Umbrella Reviews 2](#_Toc135228041)

[2. Data extraction form 6](#_Toc135228042)

[3. Codebook based on the second version of the Consolidated Framework for Implementation Research (CFIR) 10](#_Toc135228043)

[4. GRADE-CERQual 29](#_Toc135228044)

# 1. JBI Critical Appraisal Checklist for Umbrella Reviews

**Critical Appraisal Checklist^[[1]](#footnote-1)^**


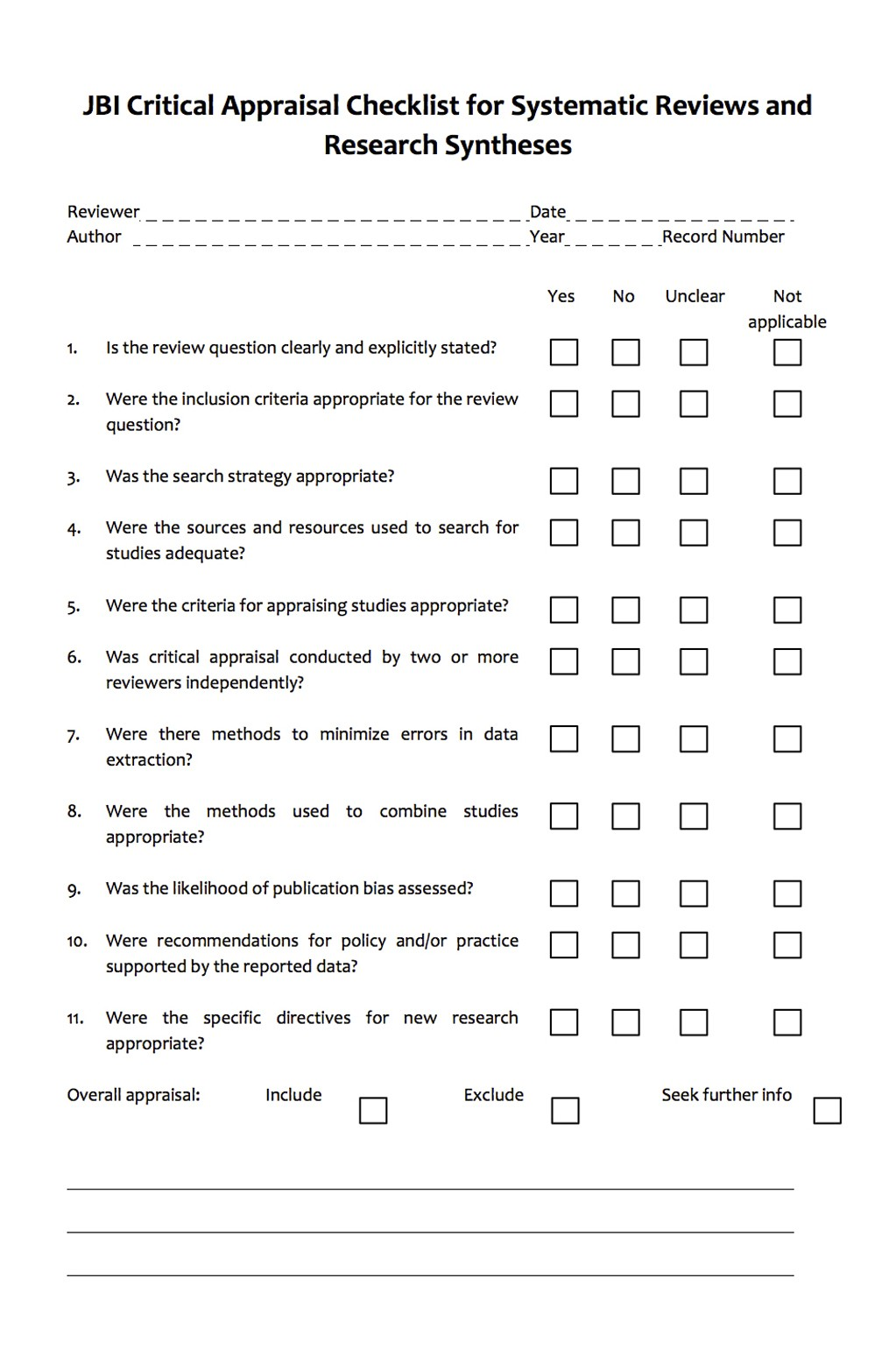


**Justification and Explanation^[[2]](#footnote-2)^**

Within Umbrella reviews, quantitative or qualitative systematic reviews may be incorporated, as well as meta-analyses of existing research. There are 11 questions to guide the appraisal of systematic reviews or meta-analyses. Each question should be answered as “yes”, “no”, or “unclear”. Not applicable “NA” is also provided as an option and may be appropriate in rare instances.

**1. Is the review question clearly and explicitly stated?**

The review question is an essential step in the systematic review process. A well-articulated question defines the scope of the review and aids in the development of the search strategy to locate the relevant evidence. An explicitly stated question, formulated around its PICO (Population, Intervention, Comparator, Outcome) elements aids both the review team in the conduct of the review and the reader in determining if they review has achieved its objectives. Ideally the review question should be articulated in a published protocol; however this will not always be the case with many reviews that are located.

**2. Were the inclusion criteria appropriate for the review question?**

The inclusion criteria should be identifiable from and match the review question. The necessary elements of the PICO should be explicit and clearly defined. The inclusion criteria should be detailed, and the included reviews should clearly be eligible when matched against the stated inclusion criteria. Appraisers of meta-analyses will find that inclusion criteria may encompass criteria around the ability to conduct statistical analyses which would not be the norm for a systematic review. The types of included studies should be relevant to the review question, for example, an Umbrella Review aiming to summarize a range of effective non-pharmacological interventions for aggressive behaviors amongst elderly patients with dementia will limit itself to including systematic reviews and meta-analyses that synthesize quantitative studies assessing the various interventions; qualitative or economic reviews would not be included.

**3. Was the search strategy appropriate?**

A systematic review should provide evidence of the search strategy that has been used to locate the evidence. This may be found in the methods section of the review report in some cases, or as an appendix that may be provided as supplementary information to the review publication. A systematic review should present a clear search strategy that addresses each of the identifiable PICO components of the review question. Some reviews may also provide a description of the approach to searching and how the terms that were ultimately used were derived, though due to limits on word counts in journals this may be more the norm in online only publications. There should be evidence of logical and relevant keywords and terms and also evidence that Subject Headings and Indexing terms have been used in the conduct of the search. Limits on the search and their potential impact should also be considered; for example, if a date limit was used, was this appropriate and/or justified? If only English language studies were included, will the language bias have an impact on the review? The response to these considerations will depend, in part, on the review question.

**4. Were the sources and resources used to search for studies adequate?**

A systematic review should attempt to identify “all” the available evidence and as such there should be evidence of a comprehensive search strategy. Multiple electronic databases should be searched including major bibliographic citation databases such as MEDLINE and CINAHL. Ideally, other databases that are relevant to the review question should also be searched, for example, a systematic review with a question about a physical therapy intervention should also look to search the PEDro database, whilst a review focusing on an educational intervention should also search the ERIC. Reviews of effectiveness should aim to search trial registries. A comprehensive search is the ideal way to minimize publication bias. As a result, a well conducted systematic review should also attempt to search for gray literature, or “unpublished” studies; this may involve searching websites relevant to the review question or thesis repositories.

**5. Were the criteria for appraising studies appropriate?**

The systematic review should present a clear statement that critical appraisal was conducted and provide details of the items that were used to assess the included studies. This may be presented in “Methods of the review”, as an appendix of supplementary information, or as a reference to a source that can be located. The tools or instruments used should be appropriate for the review question asked and the type of research conducted. For example, a systematic review of effectiveness should present a tool or instrument that addresses aspects of validity for experimental studies and randomized controlled trials such as randomization and blinding – if the review includes observational research to answer the same question, a different tool would be more appropriate. Similarly, a review assessing diagnostic test accuracy may refer to the recognized QUADAS tool (Whiting et al, 2003).

**6. Was critical appraisal conducted by two or more reviewers independently?**

Critical appraisal or some similar assessment of the quality of the literature included in a systematic review is essential. A key characteristic to minimize bias or systematic error in the conduct of a systematic review is to have the critical appraisal of the included studies completed by members of the review team independently and in duplicate. The systematic review should present a clear statement that critical appraisal was conducted by at least two reviewers working independently from each other and conferring where necessary to reach a decision regarding study quality and eligibility on the basis of quality.

**7. Were there methods to minimize errors in data extraction?**

Efforts made by review authors during data extraction can also minimize bias or systematic errors in the conduct of a systematic review. Strategies to minimize bias may include conducting all data extraction in duplicate and independently, using specific tools or instruments to guide data extraction and some evidence of piloting or training around their use.

**8. Were the methods used to combine studies appropriate?**

A synthesis of the evidence is a key feature of a systematic review. The synthesis that is presented should be appropriate for the review question and the stated type of systematic review and evidence it refers to. If a meta-analysis has been conducted this needs to be reviewed carefully. Was it appropriate to combine the studies? Have the reviewers assessed heterogeneity statistically and provided some explanation for heterogeneity that may be present? Often, where heterogeneous studies are included in the systematic review, narrative synthesis will be an appropriate method for presenting the results of multiple studies. If a qualitative review, are the methods that have been used to synthesize findings congruent with the stated methodology of the review? Is there adequate descriptive and explanatory information to support the final synthesized findings that have been constructed from the findings sourced from the original research?

**9. Was the likelihood of publication bias assessed?**

As mentioned, a comprehensive search strategy is the best means by which a review author may alleviate the impact of publication bias on the results of the review. Reviews may also present statistical tests such as Egger’s test or funnel plots to also assess the potential presence of publication bias and its potential impact on the results of the review. This question should be considered N/A for JBI qualitative reviews.

**10. Were recommendations for policy and/or practice supported by the reported data?**

Whilst the first nine questions specifically look to identify potential bias in the conduct of a systematic review, the final questions are more indicators of review quality rather than validity. Ideally a review should present recommendations for policy and practice.  Where these recommendations are made there should be a clear link to the results of the review. Is there evidence that the strength of the findings and the quality of the research have been considered in the formulation of review recommendations?

**11. Were the specific directives for new research appropriate?**

The systematic review process is recognized for its ability to identify gaps in the research, or knowledge base, around a particular topic. Most systematic review authors will provide some indication, often in the discussion section of the report, of future research direction. Where evidence is scarce or sample sizes that support overall estimates of effect are small and effect estimates are imprecise, repeating similar research to those identified by the review may be necessary and appropriate. In other instances, the case for new research questions to investigate the topic may be warranted.

# 2. Data extraction form

Notes on using a data extraction form:

- Be consistent in the order and style you use to describe the information for each included study.
- Record any missing information as unclear or not described, to make it clear that the information was not found in the study report(s), not that you forgot to extract it.
- Include any instructions and decision rules on the data collection form, or in an accompanying document. It is important to practice using the form and give training to any other authors using the form.
- You will need to protect the document in order to use the form fields (Tools / Protect document)

| **Review title or ID** |
| --- |
|  |

| **Review ID** *(surname of first author and year first full report of study published e.g. Smith 2001)* |
| --- |
|  |

| **Report IDs of other reports of this study** *(e.g. duplicate publications, follow-up studies)* |
| --- |
|  |

| **Notes:** |
| --- |

**General Information**

| **Date form completed *(dd/mm/yyyy)*** | |  |
| --- | --- | --- |
| **Name/ID of person extracting data** | |  |
| **Report title**  *(title of paper/ abstract/ report that data are extracted from)* | |  |
| **Covidence report ID**  *(if there are multiple reports of this study)* | |  |
| **Reference details** | |  |
| **Report author contact details** | |  |
| **Publication type**  *(e.g. full report, abstract, letter)* | |  |
| **Study funding source**  *(including role of funders)* | |  |
| **Possible conflicts of interest**  *(for study authors)* | |  |
| **Notes:** |  | |

**Methodological characteristics of review**

|  | | **Description** | **Location in text**  *(pg & ¶/fig/table)* |
| --- | --- | --- | --- |
| **Review aims and objectives** | |  |  |
| **Focus of the review**  *(e.g., barriers and facilitators, stakeholder perspectives)* | |  |  |
| **Context**  *(i.e., from which healthcare settings)* | |  |  |
| **Population**  *(i.e., from which study participants are drawn)* | |  |  |
| **Inclusion criteria** | |  |  |
| **Exclusion criteria** | |  |  |
| **Review type**  *(e.g., systematic review, scoping review)* | |  |  |
| **Review methodology**  *(e.g., type of synthesis method)* | |  |  |
| **Data sources** | |  |  |
| **Dates of search** | |  |  |
| **Number of included studies** | |  |  |
| **Appraisal instruments used** | |  |  |
| **Appraisal rating** | |  |  |
| **Notes:** |  | | |

**Characteristics of studies included in the review**

| **Lead author and country** | **Year of publication** | **Journal** | **Study type** | **Focus of the study** |
| --- | --- | --- | --- | --- |
|  |  |  |  |  |
|  |  |  |  |  |
|  |  |  |  |  |
|  |  |  |  |  |
|  |  |  |  |  |
|  |  |  |  |  |
|  |  |  |  |  |
|  |  |  |  |  |
|  |  |  |  |  |
|  |  |  |  |  |
|  |  |  |  |  |
|  |  |  |  |  |
|  |  |  |  |  |
|  |  |  |  |  |
|  |  |  |  |  |
|  |  |  |  |  |
|  |  |  |  |  |
|  |  |  |  |  |
|  |  |  |  |  |
|  |  |  |  |  |
|  |  |  |  |  |
|  |  |  |  |  |
|  |  |  |  |  |
|  |  |  |  |  |
|  |  |  |  |  |
|  |  |  |  |  |
|  |  |  |  |  |
|  |  |  |  |  |
|  |  |  |  |  |
|  |  |  |  |  |
|  |  |  |  |  |
|  |  |  |  |  |
|  |  |  |  |  |
|  |  |  |  |  |
|  |  |  |  |  |
|  |  |  |  |  |
|  |  |  |  |  |
|  |  |  |  |  |
|  |  |  |  |  |
|  |  |  |  |  |
|  |  |  |  |  |
|  |  |  |  |  |
|  |  |  |  |  |
|  |  |  |  |  |
|  |  |  |  |  |
|  |  |  |  |  |
|  |  |  |  |  |
|  |  |  |  |  |
|  |  |  |  |  |
|  |  |  |  |  |
|  |  |  |  |  |
|  |  |  |  |  |

**Results and other information**

|  | | **Description as stated in report/paper** | **Location in text**  *(pg & ¶/fig/table)* |
| --- | --- | --- | --- |
| **Results/findings** | |  |  |
| **Significance/direction** | |  |  |
| **Heterogeneity** | |  |  |
| **Correspondence required for further review information**  *(what and from whom)* | |  | |
| **Further review information requested**  *(from whom, what and when)* | |  | |
| **Correspondence received**  *(from whom, what and when)* | |  | |
| **Notes:** |  | | |

# 3. Codebook based on the second version of the Consolidated Framework for Implementation Research (CFIR)

**Summary table of CFIR domains, constructs and sub-constructs.^^[[3]](#footnote-3)^^**

| **Innovation Domain** | **Outer Setting Domain** | **Inner Setting Domain** | **Individuals Domain** | **Implementation Process Domain** |
| --- | --- | --- | --- | --- |
| A. Source | A. Critical Indidents | A. Structural Characteristics | ***Roles Subdomain*** | A. Teaming |
| B. Evidence-Base | B. Local Attitudes | 1. Physical Infrastructure | A. High-Level Leaders | B. Assessing Needs |
| C. Relative Advantage | C. Local Conditions | 2. Information technology Infrastructure | B. Mid-Level Leaders | 1. Innovation Deliverers |
| D. Adaptability | D. Partnerships & Connections | 3. Work Infrastructure | C. Opinion Leaders | 2. Innovation Recipients |
| E. Trialability | E. Policies & Laws | B. Relational Connections | D. Implementation Facilitators | C. Assessing Context |
| F. Complexity | F. Financing | C. Communications | E. Implementation Leads | D. Planning |
| G. Design | G. External Pressure | D. Culture | F. Implementation Team Members | E. Tailoring Strategies |
| H. Cost | 1. Societal Pressure | 1. Human-Equality Centeredness | G. Other Implementation Support | F. Engaging |
|  | 2. Market Pressure | 2. Recipient-Centeredness | H. Innovation Deliverers | 1. Innovation Deliverers |
|  | 3. Performance-Measurement Pressure | 3. Deliverer-Centeredness | I. Innovation Recipients | 2. Innovation Recipients |
|  |  | 4. Learning-Centeredness | ***Characteristics Subdomain*** | G. Doing |
|  |  | E. Tension for Change | A. Need | H. Reflecting & Evaluating |
|  |  | F. Compatibility | B. Capability | 1. Implementation |
|  |  | G. Relative Priority | C. Opportunity | 2. Innovation |
|  |  | H. Incentive Systems | D. Motivation | I. Adapting |
|  |  | I. Mission Alignment |  |  |
|  |  | J. Available Resources |  |  |
|  |  | 1. Funding |  |  |
|  |  | 2. Space |  |  |
|  |  | 3. Materials & Equipment |  |  |
|  |  | K. Access to Knowledge & Information |  |  |

**CFIR Codebook.^[[4]](#footnote-4)^**

| 1. **INNOVATION DOMAIN**   **Innovation: The “thing” being implemented, e.g., a new clinical treatment, educational program, or city service.** | |
| --- | --- |
| 1. Innovation Source | Definition: Perception of key stakeholders about whether the innovation is externally or internally developed.  Inclusion Criteria: Include statements about the source of the innovation and the extent to which interviewees view the change as internal to the organization, e.g., an internally developed program, or external to the organization, e.g., a program coming from the outside. Note: May code and rate as "I" for internal or "E" for external.  Exclusion Criteria: Exclude or double code statements related to who participated in the decision process to implement the innovation to [Engaging](http://cfirwiki.net/wiki/index.php?title=Engaging), as an indication of early (or late) engagement. Participation in decision-making is an effective engagement strategy to help people feel ownership of the innovation. |
| 1. Innovation Evidence-Base | Definition: Stakeholders’ perceptions of the quality and validity of evidence supporting the belief that the innovation will have desired outcomes.  Inclusion Criteria: Include statements regarding awareness of evidence and the strength and quality of evidence, as well as the absence of evidence or a desire for different types of evidence, such as pilot results instead of evidence from the literature.  Exclusion Criteria: Exclude or double code statements regarding the receipt of evidence as an engagement strategy to [Engaging](http://cfirwiki.net/wiki/index.php?title=Engaging): Key Stakeholders.  Exclude or double code descriptions of use of results from local or regional pilots to [Trialability](http://cfirwiki.net/wiki/index.php?title=Trialability). |
| 1. Innovation Relative Advantage | Definition: Stakeholders’ perception of the advantage of implementing the innovation versus an alternative solution.  Inclusion Criteria: Include statements that demonstrate the innovation is better (or worse) than existing programs.  Exclusion Criteria: Exclude statements that demonstrate a strong need for the innovation and/or that the current situation is untenable and code to [Tension for Change](http://cfirwiki.net/wiki/index.php?title=Tension_for_Change). |
| 1. Innovation Adaptability | Definition: The degree to which an innovation can be adapted, tailored, refined, or reinvented to meet local needs.  Inclusion Criteria: Include statements regarding the (in)ability to adapt the innovation to their context, e.g., complaints about the rigidity of the protocol. Suggestions for improvement can be captured in this code but should not be included in the rating process, unless it is clear that the participant feels the change is needed but that the program cannot be adapted. However, it may be possible to infer that a large number of suggestions for improvement demonstrates lack of compatibility, see exclusion criteria below.  Exclusion Criteria: Exclude or double code statements that the innovation did or did not need to be adapted to [Compatibility](http://cfirwiki.net/wiki/index.php?title=Compatibility). |
| 1. Innovation Trialability | Definition: The ability to test the innovation on a small scale in the organization, and to be able to reverse course (undo implementation) if warranted.  Inclusion Criteria: Include statements related to whether the site piloted the innovation in the past or has plans to in the future, and comments about whether they believe it is (im)possible to conduct a pilot.  Exclusion Criteria: Exclude or double code descriptions of use of results from local or regional pilots to [Evidence Strength & Quality](http://cfirwiki.net/wiki/index.php?title=Evidence_Strength_%26_Quality). |
| 1. Innovation Complexity | Definition: Perceived difficulty of the innovation, reflected by duration, scope, radicalness, disruptiveness, centrality, and intricacy and number of steps required to implement.  Inclusion Criteria: Code statements regarding the complexity of the innovation itself.  Exclusion Criteria: Exclude statements regarding the complexity of implementation and code to the appropriate CFIR code, e.g., difficulties related to space are coded to Available Resources and difficulties related to engaging participants in a new program are coded to [Engaging](http://cfirwiki.net/wiki/index.php?title=Engaging): Innovation Participants. |
| 1. Innovation Design | Definition: Perceived excellence in how the innovation is bundled, presented, and assembled.  Inclusion Criteria: Include statements regarding the quality of the materials and packaging.  Exclusion Criteria: Exclude statements regarding the presence or absence of materials and code to [Available Resources](http://cfirwiki.net/wiki/index.php?title=Available_Resources).  Exclude statements regarding the receipt of materials as an engagement strategy and code to [Engaging](http://cfirwiki.net/wiki/index.php?title=Engaging). |
| 1. Innnovation Cost | Definition: Costs of the innovation and costs associated with implementing the innovation including investment, supply, and opportunity costs.  Inclusion Criteria: Include statements related to the cost of the innovation and its implementation.  Exclusion Criteria: Exclude statements related to physical space and time, and code to [Available Resources](http://cfirwiki.net/wiki/index.php?title=Available_Resources). In a research study, exclude statements related to costs of conducting the research components (e.g., funding for research staff, participant incentives). |
| 1. **OUTER SETTING DOMAIN**   **Outer Setting: The setting in which the Inner Setting exists, e.g., hospital system, school district, state. There may be multiple Outer Settings and/or multiple levels within the Outer Setting (e.g., community, system, state).** | |
| 1. Critical Indidents | Definition: Large-scale and/or unanticipated events disrupt implementation and/or delivery of the innovation.  Inclusion Criteria: Include statements regarding large-scale and/or unanticipated events can disrupt implementation and/or delivery of the innovation and may include pandemics, weather-related disasters, or political disruptions.  Exclusion Criteria: TBD |
| 1. Local Attitudes | Definition: Sociocultural values (e.g., shared responsibility in helping recipients) and beliefs (e.g., convictions about the worthiness of recipients) encourage the Outer Setting to support implementation and/or delivery of the innovation.  Inclusion Criteria: Include statements regarding attitudes in the local community, which can encourage/discourage the Outer Setting to support implementation and/or delivery of the innovation. Local attitudes can be related to values (e.g., equity) and beliefs (e.g., white supremacy, racial bias); examples include widespread attitudes about organ donation and bias against tenants with housing choice vouchers.  Exclusion Criteria: TBD |
| 1. Local Conditions | Definition: Economic, environmental, political, and/or technological conditions enable the Outer Setting to support implementation and/or delivery of the innovation.  Inclusion Criteria: Include statements regarding the conditions of the local community that can enable/impede the Outer Setting to support implementation and/or delivery of the innovation. Conditions can include economic (e.g., recession), environmental (e.g., built environment), political (e.g., instability or corruption), and/or technological (e.g., IT infrastructure) factors.  Exclusion Criteria: TBD |
| 1. Partnerships & connections | Definition: The Inner Setting is networked with external entities, including referral networks, academic affiliations, and professional organization networks.  Inclusion Criteria: Include statements regarding linkages between the Inner Setting and entities in the Outer Setting, including partnerships, collaboratives, professional societies, referral networks between health and social services, community-academic partnerships, advocacy groups, contracts, technical assistance organizations, and access to regional data warehouses or membership in systems (e.g., integrated healthcare system, school district).  Exclusion Criteria: Exclude statements about general networking, communication, and relationships in the organization, such as descriptions of meetings, email groups, or other methods of keeping people connected and informed, and statements related to team formation, quality, and functioning, and code to [Networks & Communications](http://cfirwiki.net/wiki/index.php?title=Networks_%26_Communications). |
| 1. Policies & Laws | Definition: Legislation, regulations, professional group guidelines and recommendations, or accreditation standards support implementation and/or delivery of the innovation.  Inclusion Criteria: Include descriptions of externally promulgated (governmental or other external entity) policies, regulations, rules, codes, mandates, recommendations, guidelines, directives, or accreditation requirements, and alignment of implementation of the innovation with these policies and laws. This construct also includes externally generated malpractice liability and continuing education requirements.  Exclusion Criteria: TBD |
| 1. Financing | Definition: Funding from external entities (e.g., grants, reimbursement) is available to implement and/or deliver the innovation.  Inclusion Criteria: Include descriptions of payment schemes, reimbursement, remuneration, grants, and donations. It also includes overall funding constraints regionally or nationally, distinct from local economic conditions (see Local Conditions).  Exclusion Criteria: TBD |
| 1. External Pressure | Definition: External pressures drive implementation and/or delivery of the innovation.  Inclusion Criteria: Use this construct to capture themes related to External Pressures that are not included in the subconstructs below.  Exclusion Criteria: |
| 1. Societal Pressure | Definition: Mass media campaigns, advocacy groups, or social movements or protests drive implementation and/or delivery of the innovation.  Inclusion Criteria: Include statements about societal and/or mass media pressure can influence implementation and/or delivery of the innovation, as can normative pressure through, e.g., professional networks. This also includes community advocacy, pressures from class action lawsuits, and consumer groups.  Exclusion Criteria: TBD |
| 1. Market Pressure | Definition: Competing with and/or imitating peer entities drives implementation and/or delivery of the innovation.  Inclusion Criteria: Include statements about pressure from peer entities, which refer to any outside entity with which the Inner Setting feels some degree of affinity or competition (e.g., market competitors, other settings in the same network, a highly regarded institution).  Exclusion Criteria: TBD |
| 1. Performance-Measurement Pressure | Definition: Quality or benchmarking metrics or established service goals drive implementation and/or delivery of the innovation.  Inclusion Criteria: Include statements about formal mechanisms of performance accountability, audit and feedback, outcome goals, benchmarking, and public reporting.  Exclusion Criteria: TBD |
| 1. **INNER SETTING DOMAIN**   **Inner Setting: The setting in which the innovation is implemented, e.g., hospital, school, city. There may be multiple Inner Settings and/or multiple levels within the Inner Setting, e.g., unit, classroom, team. Project Inner Setting(s): [Document the actual Inner Setting in the project, e.g., type, location, and the boundary between the Outer Setting and the Inner Setting.]** | |
| **Note:** | **Constructs A – D exist in the Inner Setting regardless of implementation and/or delivery of the innovation, i.e., they are persistent general characteristics of the Inner Setting** |
| 1. Structural Characteristics | Definition: Infrastructure components support functional performance of the Inner Setting.  Inclusion Criteria: Note: Use this construct to capture themes related to Structural Characteristics that are not included in the subconstructs below.  Exclusion Criteria: |
| - 1. Physical Infrastructure | Definition: Layout and configuration of space and other tangible material features support functional performance of the Inner Setting.  Inclusion Criteria: Include descriptions of layout and configuration of space and other tangible material features can support or hinder functional performance of the Inner Setting .  Exclusion Criteria: TBD |
| - 1. Information Technology Infrastructure | Definition: Technological systems for tele-communication, electronic documentation, and data storage, management, reporting, and analysis support functional performance of the Inner Setting.  Inclusion Criteria: Include statements about information technology infrastructure.  Exclusion Criteria: TBD |
| - 1. Work infrastructure | Definition: Organization of tasks and responsibilities within and between individuals and teams, and general staffing levels, support functional performance of the Inner Setting.  Inclusion Criteria: Include descriptions of the “arrangement of tasks, responsibilities, and resources within and between the various teams in […] settings, and delegation of tasks among supervisors and subordinates.” This includes “the arrangement of schedules, shifts, and on-call duties, the order of work tasks and procedures, and the management of workloads.” General staffing (e.g., chronic understaffing) and turnover are included in this construct because of their significant effects on task allocation. General staffing levels indicate level of scarce (relatively limited) or slack (relatively abundant) resources in terms of time availability to take on new implementation.  Exclusion Criteria: TBD |
| 1. Relational Connections | Definition: There are high quality formal and informal relationships, networks, and teams within and across Inner Setting boundaries (e.g., structural, professional).  Inclusion Criteria: Include statements about general networking and relationships in the organization, such as descriptions of meetings, email groups.  Exclusion Criteria: Exclude statements related to implementation leaders' and users' access to knowledge and information regarding using the program, i.e., training on the mechanics of the program and code to [Access to Knowledge & Information](http://cfirwiki.net/wiki/index.php?title=Access_to_Knowledge_%26_Information).  Exclude statements related to engagement strategies and outcomes, e.g., how key stakeholders became engaged with the innovation and what their role is in implementation, and code to [Engaging](http://cfirwiki.net/wiki/index.php?title=Engaging): Key Stakeholders.  Exclude descriptions of outside group memberships and networking done outside the organization and code to [Cosmopolitanism](http://cfirwiki.net/wiki/index.php?title=Cosmopolitanism). |
| 1. Communications | Definition: There are high quality formal and informal information sharing practices within and across Inner Setting boundaries (e.g., structural, professional).  Inclusion Criteria: Include statements about intraorganizational communication. Making staff feel welcome (good assimilation through communication), peer collaboration, open feedback and review among peers and across hierarchical levels, clear communication of mission and goals, and informal communication quality all contribute to effective implementation.  Exclusion Criteria: TBD |
| 1. Culture | Definition: There are shared values, beliefs, and norms across the Inner Setting.  Inclusion Criteria: Note: Use this construct to capture themes related to Culture that are not included in the subconstructs below.  Exclusion Criteria: |
| - 1. Human Equality-Centeredness | Definition: There are shared values, beliefs, and norms about the inherent equal worth and value of all human beings.  Inclusion Criteria: Include statements regarding equity; shared values, beliefs, and norms about the inherent equal worth and value of all human beings may contribute to implementation outcomes as well as equity in implementation. Justice in the Inner Setting is based on perceptions of distributive and procedural fairness.  Exclusion Criteria: TBD |
| - 1. Recipient-Centeredness | Definition: There are shared values, beliefs, and norms around caring, supporting, and addressing the needs and welfare of recipients.  Inclusion Criteria: Include statements regarding six elements that can help guide evaluation of the extent to which patients are at the center of organizational processes and decisions:  patient choices are provided, patient barriers are addressed, transition between program elements is seamless, complexity and costs are minimized, and patients have high satisfaction with service and access, and receive feedback.  Exclusion Criteria: TBD |
| - 1. Deliverer-Centeredness | Definition: There are shared values, beliefs, and norms around caring, supporting, and addressing the needs and welfare of deliverers.  Inclusion Criteria: Include statements about addressing the needs of deliverers, and aligning with the expansion of the “Triple Aim” (enhancing patient experience, improving population health, reducing costs) to the “Quadruple Aim,” which adds an aim to improve the work-life and well-being of clinicians and staff.  Exclusion Criteria: TBD |
| - 1. Learning-Centeredness | Definition: There are shared values, beliefs, and norms around psychological safety, continual improvement, and using data to inform practice.  Inclusion Criteria: Include statements regarding learning culture, sychological safety, continual improvement, and using data to inform practice. Support and enable employee skill development through increased experience implementing innovations.  Exclusion Criteria: TBD |
| **Note:** | **Constructs E – K are specific to the implementation and/or delivery of the innovation.** |
| 1. Tension for Change | Definition: The current situation is intolerable and needs to change.  Inclusion Criteria: Include statements that (do not) demonstrate a strong need for the innovation and/or that the current situation is untenable, e.g., statements that the innovation is absolutely necessary or that the innovation is redundant with other programs. Note: If a participant states that the innovation is redundant with a preferred existing program, (double) code lack of [Relative Advantage](http://cfirwiki.net/wiki/index.php?title=Relative_Advantage), see exclusion criteria below.  Exclusion Criteria: Exclude statements regarding specific needs of individuals that demonstrate a need for the innovation, but do not necessarily represent a strong need or an untenable status quo, and code to [Needs and Resources of Those Served by the Organization.](http://cfirwiki.net/wiki/index.php?title=Patient_Needs_%26_Resources)  Exclude statements that demonstrate the innovation is better (or worse) than existing programs and code to [Relative Advantage](http://cfirwiki.net/wiki/index.php?title=Relative_Advantage). |
| 1. Compatibility | Definition: The innovation fits with workflows, systems, and processes.  Inclusion Criteria: Include statements that demonstrate the level of compatibility the innovation has with organizational values and work processes. Include statements that the innovation did or did not need to be adapted as evidence of compatibility or lack of compatibility.  Exclusion Criteria: Exclude or double code statements regarding the priority of the innovation based on compatibility with organizational values to [Relative Priority](http://cfirwiki.net/wiki/index.php?title=Relative_Priority), e.g., if an innovation is not prioritized because it is not compatible with organizational values. |
| 1. Relative Priority | Definition: Individuals’ shared perception of the importance of the implementation within the organization.  Inclusion Criteria: Include statements that reflect the relative priority of the innovation, e.g., statements related to change fatigue in the organization due to implementation of many other programs.  Exclusion Criteria: Exclude or double code statements regarding the priority of the innovation based on compatibility with organizational values to [Compatibility](http://cfirwiki.net/wiki/index.php?title=Compatibility), e.g., if an innovation is not prioritized because it is not compatible with organizational values. |
| 1. Incentive Systems | Definition: Tangible and/or intangible incentives and rewards and/or disincentives and punishments support implementation and delivery of the innovation.  Inclusion Criteria: Include statements related to whether organizational incentive systems are in place to foster (or hinder) implementation, e.g., rewards or disincentives for staff engaging in the innovation.  Exclusion Criteria: |
| 1. Mission Alignment | Definition: Implementing and delivering the innovation is in line with the overarching commitment, purpose, or goals in the Inner Setting.  Inclusion Criteria: Include statements related to the (lack of) alignment of implementation and innovation goals with larger organizational goals, as well as feedback to staff regarding those goals, e.g., regular audit and feedback showing any gaps between the current organizational status and the goal. Goals and Feedback include organizational processes and supporting structures independent of the implementation process. Evidence of the integration of evaluation components used as part of “Reflecting and Evaluating” into **on-going or sustained** organizational structures and processes may be (double) coded to Goals and Feedback.  Exclusion Criteria: Exclude statements that refer to the implementation team’s (lack of) assessment of the progress toward and impact of implementation, as well as the interpretation of outcomes related to implementation, and code to [Reflecting & Evaluating](http://cfirwiki.net/wiki/index.php?title=Reflecting_%26_Evaluating). Reflecting and Evaluating is part of the implementation process; it likely ends when implementation activities end. It does not require goals be explicitly articulated; it can focus on descriptions of the current state with real-time judgment, though there may be an implied goal (e.g., we need to implement the innovation) when the implementation team discusses feedback in terms of adjustments needed to complete implementation. |
| 1. Available Resources | Definition: Resources are available to implement and deliver the innovation.  Inclusion Criteria: Note: Use this construct to capture themes related to Available Resources that are not included in the subconstructs below.  Exclusion Criteria: TBD |
| - 1. Funding | Definition: Funding is available to implement and deliver the innovation financial resources is a partial mediator between management support and implementation policy and procedures.  Inclusion Criteria: Include statements regarding financial resources.  Exclusion Criteria: TBD |
| - 1. Space | Definition: Physical space is available to implement and deliver the innovation.  Inclusion Criteria: Include statements regarding “the presence/absence, design, maintenance, and allocation of areas that are properly equipped, clean, and of sufficient size and number for the provision of health care in a facility” (see also Structural Characteristics: Physical Infrastructure).  Exclusion Criteria: TBD |
| - 1. Materials & Equipment | Definition: Supplies are available to implement and deliver the innovation.  Inclusion Criteria: TBD  Exclusion Criteria: TBD |
| 1. Access to Knowledge & Information | Definition: Ease of access to digestible information and knowledge about the innovation and how to incorporate it into work tasks.  Inclusion Criteria: Include statements related to implementation leaders' and users' access to knowledge and information regarding use of the program, i.e., training on the mechanics of the program.  Exclusion Criteria: Exclude statements related to engagement strategies and outcomes, e.g., how key stakeholders became engaged with the innovation and what their role is in implementation, and code to [Engaging](http://cfirwiki.net/wiki/index.php?title=Engaging): Key Stakeholders.  Exclude statements about general networking, communication, and relationships in the organization, such as descriptions of meetings, email groups, or other methods of keeping people connected and informed, and statements related to team formation, quality, and functioning, and code to [Networks & Communications](http://cfirwiki.net/wiki/index.php?title=Networks_%26_Communications). |
| 1. **INDIVIDUALS DOMAIN**   **Individuals: The roles and characteristics of individuals.** | |
| **Roles Subdomain** | **Document the roles applicable to the project and their location in the Inner or Outer Setting.** |
| 1. High-Level Leaders | Definition: Individuals with a high level of authority, including key decision-makers, executive leaders, or directors.  Inclusion Criteria: High-level Leaders include leaders with the authority to dedicate resources and to make decisions about whether to adopt, implement, and or/sustain the innovation (see Opportunity).  Exclusion Criteria: TBD |
| 1. Mid-level Leaders | Definition: Individuals with a moderate level of authority, including leaders supervised by a high-level leader and who supervise others.  Inclusion Criteria: Mid-level managers are a key link between strategic decisions from High-level Leaders and the people who must execute implementation and accomplish delivery of the innovation. Mid-level Leaders can include clinical leaders who often lead implementation efforts and/or direct supervisors of Implementation Leads and others involved in implementation. Mid-level leaders are benefited by having skills in mediating between high-level strategy in the Inner Setting and day-to-day activities and in diffusing, selling, and synthesizing information related to the innovation and its implementation (see Capability). These leaders are more likely to support implementation if they believe that doing so will promote their own Inner Setting goals (see also Inner Setting: Mission Alignment) and if they feel involved in discussions about the implementation.  Exclusion Criteria: TBD |
| 1. Opinion Leaders | Definition: Individuals with informal influence on the attitudes and behaviors of others.  Inclusion Criteria: Opinion Leaders have informal influence on the attitudes and behaviors of people involved with implementing or delivering the innovation.  Exclusion Criteria: TBD |
| 1. Implementation Facilitators | Definition: Individuals with subject matter expertise who assist, coach, or support implementation.  Inclusion Criteria: Implementation Facilitators are individuals with subject matter expertise who assist, mentor, coach, or support implementation. Implementation Facilitators may include any individual who provides guidance to the Implementation Leads or Teams. Implementation Facilitators can play an integral role throughout implementation, formally influencing or facilitating innovation decisions in a desirable direction. External facilitators usually have professional training in a technical field related to organizational change science or in the technology being introduced into the organization. This role includes outside researchers who may be implementing a multi-site innovation study and other formally appointed individuals from the Outer Setting, e.g., a facilitator from a corporate or regional office or a hired consultant.  Exclusion Criteria: TBD |
| 1. Implementation Leads | Definition: Individuals who lead efforts to implement the innovation.  Inclusion Criteria: Implementation Leads may emerge organically out of a grassroots (bottom-up) initiative to e.g., improve use of an innovation. Alternatively, Implementation Leads may be identified through top-down assignment. Ilot et al. found that “none of the instigators” of implementation were formally appointed in their cross-case comparison study, though some ultimately assumed that role. Individuals who volunteer to lead may be more effective than those who were assigned the role.  Exclusion Criteria: TBD |
| 1. Implementation Team Members | Definition: Individuals who collaborate with and support the Implementation Leads to implement the innovation, ideally including Innovation Deliverers and Recipients.  Inclusion Criteria: Implementation Team Members include individuals who directly or indirectly participate in implementation and support the Implementation Leads. Implementation teams can play a critical role in implementation, because Implementation Leads are not as effective alone.  Exclusion Criteria: TBD |
| 1. Other Implementation Support | Definition: Individuals who support the Implementation Leads and/or Implementation Team Members to implement the innovation.  Inclusion Criteria: Other key roles in implementation include individuals who assist the Implementation Leads and Team Members with implementation. These individuals can perform many different functions, including providing technical assistance for information technology, human resources, contracting, etc. It can also include integrators who help to build relationships between Inner Settings.  Exclusion Criteria: TBD |
| 1. Innovation Deliverers | Definition: Individuals who are directly or indirectly delivering the innovation.  Inclusion Criteria: Includes administrators, clinicians, and others who deliver or support delivery of the innovation (within and outside the Inner Setting).  Exclusion Criteria: TBD |
| 1. Innovation Recipients | Definition: Individuals who are directly or indirectly receiving the innovation  Inclusion Criteria: Innovation Recipients include anyone expected to benefit from implementation of the innovation. It is important to center recipients to help ensure their needs (see Process: Assessing Needs) are prioritized (see Inner Setting: Culture-Recipient-centeredness). Types of recipients have included, but are not limited to, community health workers, outreach teams, nurses, etc. Other terms used for Innovation Recipients have included consumers or clients. Innovation Recipients are determined by the goals and focus for implementing an innovation.  Exclusion Criteria: TBD |
| **Characteristics Subdomain** | **Project Characteristics: [Document the characteristics applicable to the roles in the project based on the COM-B system or role-specific theories.]** |
| 1. Need | Definition: The individual(s) has deficits related to survival, well-being, or personal fulfillment, which will be addressed by implementation and/or delivery of the innovation.  Inclusion Criteria: Aims, wishes, and needs are important to assess for all constituents (see also Process: Assessing Needs), and the level of awareness of recipient and deliverer needs is an important implementation determinant. Within healthcare delivery settings, consideration of patient needs must be integral to any implementation that seeks to improve patient outcomes.  Exclusion Criteria: TBD |
| 1. Capability | Definition: The individual(s) has interpersonal competence, knowledge, and skills to fulfill Role.  Inclusion Criteria: Includes psychological and physical ability. Intrapersonal competence, knowledge, and skills to fulfill roles are important for successful implementation. Past experiences with implementation and/or the innovation helps build capability. Capabilities including personal traits of competence and learning style are important for tailoring training strategies.  Exclusion Criteria: TBD |
| 1. Opportunity | Definition: The individual(s) has availability, scope, and power to fulfill Role.  Inclusion Criteria: Includes “all the factors that lie outside the individual that make the behavior possible or prompt it”. In the updated CFIR, themes related to this construct are specific to implementing and delivering an innovation in the Inner Setting, including staff availability and sufficient time allocation, autonomy, and control to fulfill the role. The quality of support provided by the Inner Setting to individuals is positively associated with implementation.  Exclusion Criteria: TBD |
| 1. Motivation | Definition: The individual(s) is committed to fulfilling Role.  Inclusion Criteria: Includes brain processes that energize and direct behavior and commitment, the act of binding oneself to a course of action intellectually and/or emotionally. This construct includes commitment of individuals to fulfill their role. Perceptions of the commitment of leaders was captured in the original CFIR as part of the Inner Setting, but the updated CFIR recognizes the importance of capturing this theme for all individual roles.  Exclusion Criteria: TBD |
| 1. **IMPLEMENTATION PROCESS DOMAIN**   **Implementation Process: The activities and strategies used to implement the innovation. Project Implementation Process: [Document the implementation process framework and/or activities and strategies being used to implement the innovation. Distinguish the implementation process used to implement the innovation (activities that end after implementation is complete) from the innovation (the “thing” that continues when implementation is complete).]** | |
| 1. Teaming | Definition: Join together, intentionally coordinating and collaborating on interdependent tasks, to implement the innovation.  Inclusion Criteria: Include statements regarding team capabilities, social relationships, teamwork, and morale in accomplishing their goals (see also Implementation Team Members and Characteristics).  Exclusion Criteria: TBD |
| 1. Assessing Needs | Definition: Collect information about priorities, preferences, and needs of people. Note: Use this construct to capture themes related to Assessing Needs that are not included in the subconstructs below.  Inclusion Criteria: Include statements regarding the assessment of the needs of both recipients and deliverers to guide the implementation process is an important determinant to implementation success as well as equity in implementation.  Exclusion Criteria: TBD |
| - 1. Innnovation Deliverers | Definition: Collect information about the priorities, preferences, and needs of deliverers to guide implementation and delivery of the innovation.  Inclusion Criteria: TBD  Exclusion Criteria: TBD |
| - 1. Innovation Recipients | Definition: Collect information about the priorities, preferences, and needs of recipients to guide implementation and delivery of the innovation.  Inclusion Criteria: TBD  Exclusion Criteria: TBD |
| 1. Assessing Context | Definition: Collect information to identify and appraise barriers and facilitators to implementation and delivery of the innovation.  Inclusion Criteria: Assessing context is foundational within implementation science and ideally utilizes a determinant framework to guide the assessment. Assessments should consider all salient contextual factors — both modifiable and non-modifiable. Workarounds can be developed for non-modifiable factors, and strategies can be targeted to modify factors (e.g., increase the knowledge of deliverers about the innovation).  Exclusion Criteria: TBD |
| 1. Planning | Definition: Identify roles and responsibilities, outline specific steps and milestones, and define goals and measures for implementation success in advance.  Inclusion Criteria: The fundamental objective of planning is to design a course of action to promote effective implementation by building local capacity for using the innovation, collectively and individually; this construct includes contingency planning, goal-setting (see also Reflecting and Evaluation for monitoring progress toward set goals), selecting strategies, and occurs within context of incremental implementation approaches or testing cycles.  Exclusion Criteria: TBD |
| 1. Tailoring Strategies | Definition: Choose and operationalize implementation strategies to address barriers, leverage facilitators, and fit context.  Inclusion Criteria: Implementation strategies are chosen and tailored to address findings from Assessing Needs and Assessing Context. There are many approaches for tailoring strategies, including implementation mapping, which can be used to operationalize chosen strategies and then reported following published guidelines.  Exclusion Criteria: TBD |
| 1. Engaging | Definition: Attracting and involving appropriate individuals in the implementation and use of the innovation through a combined strategy of social marketing, education, role modeling, training, and other similar activities.  Inclusion Criteria: Note: Use this construct to capture themes related to Engaging that are not included in the subconstructs below.  Exclusion Criteria: Exclude statements related to specific sub constructs, e.g., [Champions](http://cfirwiki.net/wiki/index.php?title=Champions) or [Opinion Leaders](http://cfirwiki.net/wiki/index.php?title=Opinion_Leaders).  Exclude or double code statements related to who participated in the decision process to implement the innovation to [Innovation Source](http://cfirwiki.net/wiki/index.php?title=Intervention_Source), as an indicator of internal or external innovation source. |
| 1. Innovation Deliverers | Definition: Attract and encourage deliverers to serve on the implementation team and/or to deliver the innovation.  Inclusion Criteria: Include statements related to engagement strategies and outcomes, i.e., if and how staff became engaged with the innovation and what their role is in implementation.  Exclusion Criteria: |
| 1. Innovation Recipients | Definition: Attract and encourage recipients to serve on the implementation team and/or participate in the innovation.  Inclusion Criteria: Include statements related to engagement strategies and outcomes, i.e., if and how innovation recipients became engaged with the innovation and what their role is in implementation.  Exclusion Criteria: |
| 1. Doing | Definition: Implement in small steps, tests, or cycles of change to trial and cumulatively optimize delivery of the innovation.  Inclusion Criteria: Include statements that demonstrate how implementation occurred with respect to the implementation plan. Note: Executing is coded very infrequently due to a lack of planning. However, some studies have used fidelity measures to assess executing, as an indication of the degree to which implementation was accomplished according to plan.  Exclusion Criteria: |
| 1. Reflecting & Evaluating | Definition: Quantitative and qualitative feedback about the progress and quality of implementation accompanied with regular personal and team debriefing about progress and experience.  Inclusion Criteria: Note: Use this construct to capture themes related to Reflecting & Evaluating that are not included in the subconstructs below.  Exclusion Criteria: Exclude statements related to the (lack of) alignment of implementation and innovation goals with larger organizational goals, as well as feedback to staff regarding those goals, e.g., regular audit and feedback showing any gaps between the current organizational status and the goal, and code to [Goals & Feedback](http://cfirwiki.net/wiki/index.php?title=Goals_%26_Feedback). Goals and Feedback include organizational processes and supporting structures independent of the implementation process. Evidence of the integration of evaluation components used as part of “Reflecting and Evaluating” into **on-going or sustained** organizational structures and processes may be (double) coded to Goals and Feedback.  Exclude statements that capture reflecting and evaluating that participants may do during the interview, for example, related to the success of the implementation, and code to [Knowledge & Beliefs about the Innovation](http://cfirwiki.net/wiki/index.php?title=Knowledge_%26_Beliefs_about_the_Intervention). |
| - 1. Implementation | Definition: Collect and discuss quantitative and qualitive information about the success of implementation.  Inclusion Criteria: This subconstruct includes tracking progress towards achieving implementation goals and milestones using rigorous and pragmatic monitoring and evaluation methods.  Exclusion Criteria: TBD |
| - 1. Innovation | Definition: Collect and discuss quantitative and qualitative information about the success of the innovation.  Inclusion Criteria: This construct includes monitoring and optimizing outcomes for key constituencies using rigorous and pragmatic monitoring and evaluating methods.  Exclusion Criteria: TBD |
| 1. Adapting | Definition: Modify the innovation and/or the Inner Setting for optimal fit and integration into work processes.  Inclusion Criteria: There is extensive literature that emphasizes the key role of adaptations to ensure optimal fit between the innovation and setting(s) within which it is being implemented and delivered. Adapting includes determining and documenting the types of adaptations made, ideally following an adaptation framework. We refer users to above cited articles for further guidance on best practices for adapting.  Exclusion Criteria: TBD |
| 1. **Additional Codes** |  |
| 1. Code Name | Definition:  Inclusion Criteria:  Exclusion Criteria: |
| 1. Code Name | Definition:  Inclusion Criteria:  Exclusion Criteria: |

**General Coding Rules:**

When two codes are in question for a passage, consider the primary meaning of the passage to assign code; consider what the participant is truly saying. Analysts may wish to err on the side of inclusion or double coding.

**General Rating Rules:**

| **Ratings** | | | | | | |
| --- | --- | --- | --- | --- | --- | --- |
| M | -2 | -1 | 0 | X | +1 | +2 |

In general, ratings are determined based on two factors: 1) valence and 2) strength.

**Valence: positive or negative influence on implementation**

*Rating component: X, 0, +, -*

The valence component of a rating is determined by the influence the coded data has on the implementation process, i.e., contextual factors that facilitate or hinder implementation. Due to limited data, analysts may have to infer the influence on implementation based on simple presence or absence of a construct. For example, if a participant states that the intervention has advantages over existing programs, but does not state how this has influenced implementation, the analyst can infer that the presence of relative advantage facilitated implementation. However, whenever the data allows, the analysts should apply ratings based on the influence the construct has on implementation, not the presence or absence of a construct; presence or absence of a positive construct (e.g. relative advantage) does not always constitute a matching positive or negative influence on implementation.

In the event that comments are mixed, i.e., some comments are negative and some comments are positive, try to tip the rating to a weak positive or weak negative, based on the aggregate of the comments. However, if you feel the comments are equally positive and negative, apply a mixed (X) rating. Some users of the CFIR have denoted level of agreement among participants in their rating by adding a * to the rating if comments were mixed. For example, if the aggregate of mixed comments was positive, the rating was +1*. Some users feel it’s important to record discord among participants because it indicates a negative influence on implementation.

In the event that the comments are neutral, i.e., comments are related to a construct but have no bearing on the implementation, apply the neutral (0) rating.

**Strength: weak or strong influence on implementation**

*Rating component: 1, 2*

The strength component of a rating is determined by a number of factors, including: level of agreement among participants, strength of language, and use of concrete examples. However, sometimes analysts may choose to apply relative ratings, versus absolute ratings, in order to differentiate between organization in the study.

# 4. GRADE-CERQual

The GRADE-CERQual (Grading of Recommendations Assessment, Development and Evaluation-Confidence in Evidence from Reviews of Qualitative research) approach has been developed by the GRADE working group.

CERQual includes four components for assessing how much confidence to place in findings from reviews of qualitative research (also referred to as qualitative evidence syntheses): (1) methodological limitations, (2) relevance, (3) coherence and (4) adequacy of data.

**Criteria for the assessment of methodological limitations^[[5]](#footnote-5)^**

In the context of this umbrella review, methodological limitations are the extent to which there are concerns about the design or conduct of the reviews that contributed evidence to an individual finding of the umbrella review. This will be assessed using the result of the JBI Critical Appraisal Checklist of each included review.

| **Result of JBI Critical Appraisal** | **Score GRADE-CERQual** |
| --- | --- |
| All questions answered by “Yes” | No to very minor concerns (4 points) |
| 1 question answered by “Unclear” or “No” | Minor concerns (3 points) |
| 2-3 questions answered by “Unclear” or “No” | Moderate concerns (2 points) |
| 4 questions or more answered by “Unclear” or “No” | Substantial concerns (1 point) |

**Criteria for the assessment of relevance**

In the context of this umbrella review, relevance is the extent to which the body of data from the included reviews supporting an individual finding of the umbrella review is applicable to the context (perspective or population, phenomenon of interest, setting) specified in the umbrella review question(s).

| **Result of relevance assessment** | **Score GRADE-CERQual** |
| --- | --- |
| Most reviews are of direct relevance | No to very minor concerns (4 points) |
| Most reviews are of indirect relevance | Minor concerns (3 points) |
| Most reviews are of partial relevance | Moderate concerns (2 points) |
| Most reviews are of uncertain relevance | Substantial concerns (1 point) |

**Criteria for the assessment of coherence**

In the context of this umbrella review, coherence is how clear and cogent the fit is between the data from the reviews and an umbrella review finding that synthesises that data. Assessment based on three criteria:

1. ***Contradictory data:*** Some of the data from included reviews *contradict* the umbrella review finding.
2. ***Ambiguous or incomplete data:*** Key aspects of the underlying data may be vaguely defined or described
3. ***Plausible alternatives:*** Plausible alternative descriptions, interpretations or explanations could be used to synthesise the underlying data.

| **Based on threats to coherence** | **Score GRADE-CERQual** |
| --- | --- |
| No threat to coherence | No to very minor concerns (4 points) |
| One threat to coherence | Minor concerns (3 points) |
| Two threats to coherence | Moderate concerns (2 points) |
| Three threats to coherence | Substantial concerns (1 point) |

**Criteria for the assessment of adequacy of data**

In the context of this umbrella review, adequacy of data is the overall determination of the degree of richness and the quantity of data supporting an umbrella review finding.

| **Based on ‘data sources’** | **Score GRADE-CERQual** |
| --- | --- |
| Extensive search/resources + defined number of articles | No to very minor concerns (4 points) |
| Extensive search/resources + no defined number of articles or limited articles | Minor concerns (3 points) |
| Limited search | Moderate concerns (2 points) |
| Not defined | Substantial concerns (1 point) |

1. Joanna Briggs Institute. (2022). Appendix 10.1 JBI Critical Appraisal Checklist for Systematic Reviews and Research Syntheses. JBI Evidence Synthesis Manual. <https://jbi-global-wiki.refined.site/space/MANUAL/4687059/Appendix+10.1+JBI+Critical+Appraisal+Checklist+for+Systematic+Reviews+and+Research+Syntheses> [↑](#footnote-ref-1)
2. Joanna Briggs Institute. (2022). Appendix 10.2. Discussion of JBI Critical Appraisal Checklist for systematic reviews and research syntheses. JBI Evidence Synthesis Manual. <https://jbi-global-wiki.refined.site/space/MANUAL/4687022/Appendix+10.2.+Discussion+of+JBI+Critical+Appraisal+Checklist+for+systematic+reviews+and+research+syntheses> [↑](#footnote-ref-2)
3. Damschroder, L.J., Reardon, C.M., Widerquist, M.A.O. *et al.* The updated Consolidated Framework for Implementation Research based on user feedback. *Implementation Sci* **17**, 75 (2022). <https://doi.org/10.1186/s13012-022-01245-0> [↑](#footnote-ref-3)
4. Damschroder, L.J., Reardon, C.M., Widerquist, M.A.O. *et al.* The updated Consolidated Framework for Implementation Research based on user feedback. *Implementation Sci* **17**, 75 (2022). <https://doi.org/10.1186/s13012-022-01245-0> [↑](#footnote-ref-4)
5. Lewin, S., Booth, A., Glenton, C. *et al.* Applying GRADE-CERQual to qualitative evidence synthesis findings: introduction to the series. *Implementation Sci* **13** (Suppl 1), 2 (2018). https://doi.org/10.1186/s13012-017-0688-3 [↑](#footnote-ref-5)
